# The Neurosurgeon’s Armamentarium for Gliomas: An Update on Intraoperative Technologies to Improve Extent of Resection

**DOI:** 10.3390/jcm10020236

**Published:** 2021-01-11

**Authors:** Alexander J. Schupper, Raymund L. Yong, Constantinos G. Hadjipanayis

**Affiliations:** Department of Neurosurgery, Mount Sinai Health System, New York, NY 10029, USA; raymund.yong@mountsinai.org (R.L.Y.); constantinos.hadjipanayis@mountsinai.org (C.G.H.)

**Keywords:** extent of resection, fluorescence-guided surgery, 5-ALA, fluorescein, intraoperative imaging, neuromonitoring, iMRI, ultrasound, glioma surgery, glioblastoma

## Abstract

Maximal safe resection is the standard of care in the neurosurgical treatment of high-grade gliomas. To aid surgeons in the operating room, adjuvant techniques and technologies centered around improving intraoperative visualization of tumor tissue have been developed. In this review, we will discuss the most advanced technologies, specifically fluorescence-guided surgery, intraoperative imaging, neuromonitoring modalities, and microscopic imaging techniques. The goal of these technologies is to improve detection of tumor tissue beyond what conventional microsurgery has permitted. We describe the various advances, the current state of the literature that have tested the utility of the different adjuvants in clinical practice, and future directions for improving intraoperative technologies.

## 1. Introduction

Maximal extent of resection has been shown to improve both progression-free survival as well as overall survival in the surgical treatment of gliomas [1,2,3]. Since many of these lesions involve or approach eloquent brain regions, there exists an important balance between maximizing cytoreduction and minimizing neurological deficit [4]. The challenge surgeons face technically is distinguishing the interface between tumor tissue and the surrounding brain. Technological advancements have led to improvements in the visual discrimination of the infiltrative tumor–brain margin in order to maximize safe resection.

The objective of this review is to outline the current advances in glioma surgery and describe the adjuncts surgeons utilize to achieve the optimal cytoreductive result while maintaining patient safety. Through the use of fluorescence-guided surgery (FGS), imaging, neuromonitoring, and novel handheld devices, these intraoperative technologies have allowed the surgeon to visualize tumor at the cellular level and perform microsurgery with increasing precision. This review will discuss the technical aspects of various intraoperative adjuncts currently available that can impact patient outcomes and cutting-edge technologies to come.

## 2. Fluorescence-Guided Surgery (FGS)

**Figure 1:**
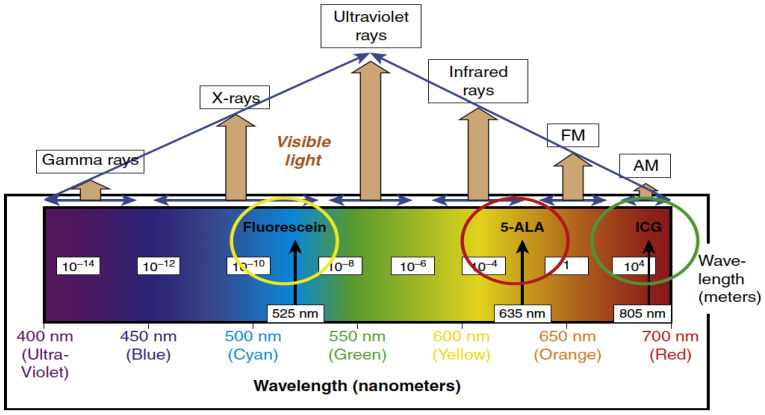
Fluorescence emission wavelengths of the most commonly used fluorophores used in glioma surgery. 5-ALA, 5-aminolevulinic acid; ICG, indocyanine green; FM, Frequency Modulation; AM, Amplitude Modulation. (Permission from Hadjianapyis CG, Stummer W. Fluorescence Guided Neurosurgery. New York: Thieme Medical Publishers; 2018) [5].

Fluorescence-guided surgery (FGS) has provided the neurosurgeon real-time intraoperative visualization of brain tumors aiding in the maximal resection of tumors. The three most common agents used during FGS in neurosurgical oncology (5-aminolevulinic acid (5-ALA), fluorescein, and indocyanine green (ICG)) are discussed below. These fluorophores emit light in both the visible and near infrared spectrum (Figure 1). This fluorescence focuses at the surface of the surgical cavity, helping surgeons distinguish not only the tumor core, but the tumor-brain interface that often dictates the extent of resection. Several techniques to be discussed later have been established to improve light penetrance, as a limitation of FGS is the surface visualization of the fluorescence, when compared to other imaging modalities, such as ultrasound and intraoperative MRI. In addition to the main fluorophores currently used in practice, other fluorophores under investigation are described, as well as the future direction for FGS. A table summarizing landmark studies for the various fluorophores may be found in Table 1.

### 2.1. 5-Aminolevulinic Acid (5-ALA)

#### 2.1.1. 5-ALA: Background and Mechanism of Action

**Figure 2:**
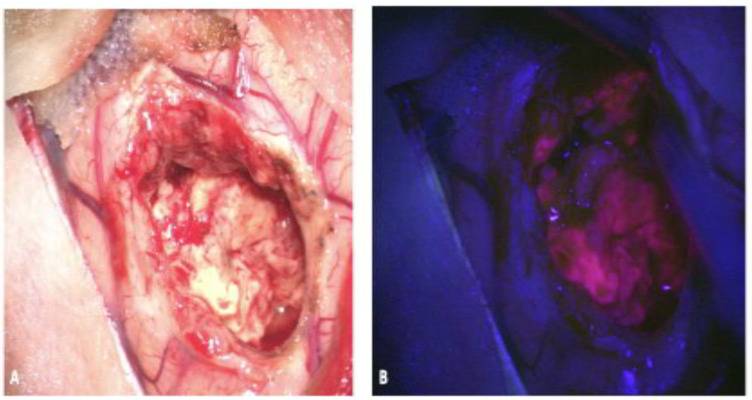
Fluorescence-guided surgery using 5-ALA for resection of a high-grade glioma. (**A**) shows the resection cavity under white light and (**B**) shows the red and pink fluorescence under blue light based upon tumor cell density, with the surrounding normal brain appearing blue without signs of fluorescence. 5-ALA, 5-aminolevulinic acid. (Permission from Hadjipanayis CG et al. Fluorescence Guided Brain Tumor Surgery. Youmans & Winn Neurological Surgery 8th Edition. Chapter 157B. New York: Elsevier; 2021) [21].

The most widely studied fluorophore used in glioma surgery is 5-aminolevulinic acid (5-ALA). Ingested orally prior to surgery, 5-ALA is converted to protoporphyrin IX (PPIX) in the heme biosynthesis pathway, and accumulates intracellularly within glioma cells. Excited by blue light in the 400 nm range, PPIX fluoresces red-violet at two emission peaks (635 and 704 nm) (Figure 2). 5-ALA is rapidly absorbed through the gastrointestinal tract into the bloodstream and is converted into PPIX within glioma cells within hours. Intracellular accumulation of PPIX has been shown to persist for at least nine hours [20].

#### 2.1.2. 5-ALA: Limitations

While 5-ALA has shown generally high sensitivity and PPV for HGG tissue in multiple studies [22], there are reports of false positive and negative fluorescence [23,24]. False positive fluorescence has been reported in regions of recurrent HGGs that may be associated with treatment effect [25]. Several studies have reported that 5-ALA has modest specificity and negative PPV which is due to the fact that fluorescence becomes difficult to detect with current visualization technology in the infiltrative margin of gliomas. As the neurosurgeon resects tumor tissue further out in the infiltrative margin, lower tumor cell density results in less or no fluorescence visualization. However, new visualization devices can permit quantification of 5-ALA (PPIX) signal intensity and detection of fluorescence [26], which correlates with tumor cell density [27], and has been also shown to correlate with Ki-67/MIB-1 index [28,29]. Furthermore, 5-ALA induced fluorescence is found with ependymal surfaces in the ventricles which in certain patients may be associated with subependymal spread of their HGG [28,30,31].

#### 2.1.3. 5-ALA: Evidence for Use

With over 40 clinical trials to date and regulatory approval in a number of countries throughout the world, 5-ALA has been established as part of the standard of care in high-grade glioma (HGG) surgery [22]. Dr. Walter Stummer first described the use of 5-ALA in 1998 where it was found to have a high sensitivity of 85% and specificity of 100% in 89 tissue biopsies [32]. Due to its selective uptake in glioma cells, 5-ALA has consistently been found in a number of studies to have high sensitivity and positive predictive value (PPV) in both new and recurrent HGG [9,22,23,28,33]. In addition to its study in HGG tumors, the use of 5-ALA has been expanded to other tumor types, including meningioma [34], brain metastases [35], primary CNS lymphoma [36], hemangioblastoma [37], ependymomas [38], subependymomas [39], and ATRT [40]. The use of 5-ALA has also recently expanded to low-grade gliomas (LGG). While initial observations by Stummer and others did not show fluorescence in LGG portions of tumors [21], in the largest series to date Jaber et al. found visible fluorescence in 59 of 76 WHO grade III gliomas, and only visible fluorescence in 13 of 82 WHO grade II gliomas. [41] Therefore, we conclude that most WHO grade III and IV gliomas show 5-ALA fluorescence, while lower grade gliomas do not.

The only randomized controlled trial of FGS has been with the use of 5-ALA. In 2006, a landmark German study where newly diagnosed HGG patients were randomized to undergo 5-ALA FGS or conventional microsurgery, found that 5-ALA FGS resulted in almost a doubling of complete resection of the contrast-enhancing portion of tumors in comparison to conventional microsurgery [7]. Furthermore, patient outcomes were better in the 5-ALA FGS group where patients had an improvement (41% versus 21%) in six-month progress-free survival (PFS-6) [7]. Patients in this study all underwent adjuvant fractionated radiation therapy and only a small portion of patients underwent adjuvant chemotherapy since the current standard of care use of concomitant temozolomide and radiotherapy followed by adjuvant temozolomide (Stupp protocol) was not yet established. Since this trial, Diez Valle et al. performed a retrospective, observational study where patients underwent 5-ALA FGS and the Stupp protocol to determine the additive effect on patient outcomes and found an even greater progression-free survival advantage (69% versus 48%) [12]. The results of the Stummer randomized controlled trial, as well other studies, served as the basis for the approval of 5-ALA by the Food and Drug Administration (FDA) in 2017 for use as an optical imaging agent to visualize malignant tumor tissue during glioma surgery [24]. Since its recent approval, 5-ALA has been widely utilized in the United States (Gleolan^©^), and the first US multicenter trial has recently been completed (NCT02632370). In a meta-analysis of 5-ALA studies for glioblastoma surgery, Eljamel found that 5-ALA FGS contributed a mean overall survival advantage of 6.2 months. However, this survival advantage may be influenced by the type and number of adjuvant treatments GBM patients undergo [13]. Moreover, 5-ALA is generally well-tolerated with minimal adverse events, most commonly skin photosensitivity in the immediate postoperative period [7] and subclinical transient elevations in liver enzymes [14].

### 2.2. Fluorescein

#### 2.2.1. Fluorescein: Background and Mechanism of Action

**Figure 3:**
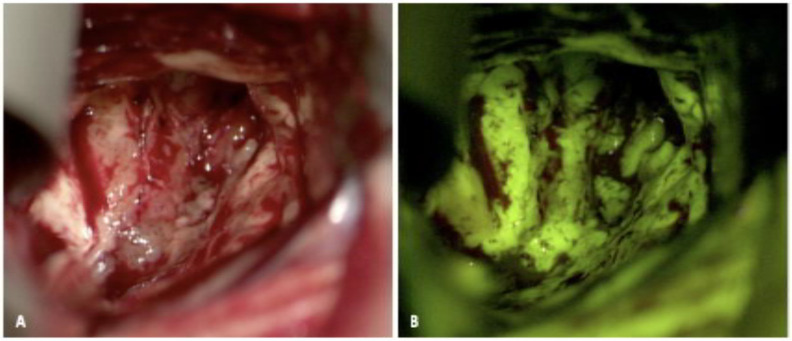
Fluorescence-guided surgery using fluorescein sodium for resection of a high-grade glioma. (**A**) shows the resection cavity under white light and (**B**) shows the green-yellow fluorescence under fluorescence. Fluorescein is not tumor-specific, as seen by the extravasated fluorophore within normal brain tissue and edematous brain. (Permission from Hadjianapyis CG et al. Fluorescence Guided Brain Tumor Surgery. Youmans & Winn Neurological Surgery 8th Edition. Chapter 157B. New York: Elsevier; 2021) [21].

Fluorescein sodium was the first fluorophore to be used to detect brain tumors. It is FDA-approved in ophthalmology and widely used in retinal exams. Dr. George E. Moore first described the use of fluorescein in identifying malignant gliomas in 1947 [42]. With a peak absorption between 465 and 480 nm and an emission peak just over 500 nm, fluorescein exhibits a yellow-green fluorescence that allows for fluorescence detection with ambient light conditions (Figure 3) [43]. It is well tolerated, with uncommon side effects such as skin and urine discoloration at high doses [44]. Unlike 5-ALA, fluorescein is given intravenously following anesthesia induction, and travels to areas of blood-brain barrier (BBB) breakdown and increased vascularity, permitting accumulation in HGGs. However, it does not accumulate intracellularly as seen with 5-ALA, but rather in the extracellular space, which leads to non-specific signal due to the fluorescence of dura, blood vessels, and any perturbed peritumoral tissue [45].

#### 2.2.2. Fluorescein: Evidence for Use

To date, there have been over 10 clinical studies on the use of fluorescein for glioma surgery [22]. A number of studies have confirmed positive extent of resection with the use of fluorescein FGS [15,43,46,47]. In 2018, a European multicenter phase II non-randomized, single-arm prospective trial (FLOUGLIO) was conducted and found that 82.6% of patient had complete resection of their contrast-enhancing tumor, or gross total resection (GTR), with a median survival of 12 months [18]. In this study, fluorescein was found to have a sensitivity and specificity of 80% for HGGs. These studies have found fluorescein to be safe and effective, with minimal associated adverse events.

Despite the increasing number of studies on its use in glioma surgery, there have been no randomized controlled trials assessing the use of fluorescein. Additionally, many studies have small sample sizes including the phase II trial, which only included 46 patients. While prior studies have shown good correlation between contrast-enhancement on preoperative MRI imaging and intraoperative fluorescence [18,46,48], the lack of any controlled studies calls the true efficacy of this fluorophore in glioma surgery into question, and further investigation is needed for stronger conclusions on its use. 

Recently, in a dual-labeling study of combined 5-ALA and fluorescein, the background fluorescence of fluorescein together with the specific intracellular signal of PpIX seemed to improve intraoperative visualization of HGG compared to conventional white light [49]. Fluorescein and 5-ALA have also previously been compared, and in a retrospective single-center study of over 200 patients receiving either 5-ALA or fluorescein for HGG resection, there were no differences in extent of resection or mean overall survival [50].

#### 2.2.3. Fluorescein: Limitations

Despite the advantages of fluorescein being widely available and cost-effective, non-specific, extracellular accumulation is a major limitation of its use. Prior studies have shown fluorescein as a marker for edema propagation [51], rather than tumor tissue itself. Additionally, as the blood–brain barrier is disrupted during cytoreduction, fluorescein extravasates, impairing the distinction of the tumor-brain interface at the infiltrative margin. Finally, extravasated fluorescein may stain normal brain and edematous tissue surrounding the tumor, which may create additional challenges with resection [52].

### 2.3. ICG

#### 2.3.1. ICG: Background and Mechanism of Action

Indocyanine green (ICG) is a well-known fluorophore that is used in a variety of medical fields including hepatology and ophthalmology [22], and is most commonly used in neurosurgery in the context of intraoperative videoangiography [53]. Unlike 5-ALA and fluorescein, ICG emits light in the near-infrared (NIR) spectrum, which allows for deeper penetration and visualization into target tissues [54]. Similar to fluorescein, ICG works by passively collecting in the extracellular compartment of tumors after intravenous injection in areas of BBB disruption. Second-window-ICG (SWIG) has recently been described, where higher doses of ICG are given the day prior to surgery, allowing accumulation of the fluorophore in brain tumors due to enhanced permeability through the endothelium, known as the enhanced permeability and retention effect, or EPR effect [55]. ICG is believed to bind to albumin intravascularly, prior to accumulating in tumors through areas of BBB breakdown [56].

#### 2.3.2. ICG: Evidence for Use

There have been several recent studies assessing the use of ICG in the resection of gliomas and other CNS tumors. Two prior clinical trials showed that ICG improves tumor visualization at the tumor margin [57,58], and Li et al. showed that NIR excitation of ICG improved the signal to background ratio (SBR) with the addition of lasers in the NIR spectrum, compared to “non-boosted” samples [59]. Recently, the second-window-ICG (SWIG) technique has been shown to have utility in gliomas, as well as meningiomas, metastatic lesions, chordomas, and other primary brain tumors [19,55]. In a small study of 15 glioma resections, Lee et al. found a sensitivity and specificity of 84% and 80%, respectively, with ICG FGS, and demonstrated strong correlation with the degree of contrast on postoperative MRI [56]. Currently, there have been no trials on the effect of ICG on patient outcomes following surgery, and ICG has not previously been shown to improve EOR [58]. ICG has been shown to be safe and well tolerated [53].

#### 2.3.3. ICG: Limitations

ICG has the potential benefit of being a NIR agent which may permit for better detection and visualization of fluorescence transdurally and in the brain. However, technical limitations of ICG fluorescence must be mentioned. The major limitation to ICG is the high false-positive detection. All gadolinium-enhancing tissue will exhibit an ICG signal, including areas of inflammation and necrosis [55]. Additionally, as the NIR spectrum is not part of the spectrum typically used in microscopic surgery, it can be difficult to operate in this range, requiring transitioning between white light and the near-infrared. In most instances, visualization of NIR fluorescence requires a separate display monitor that only displays the ICG fluorescence and a dark background that is difficult to distinguish surrounding brain structures. In order to use ICG for FGS, tumor tissue fluorescence must be overlaid on the standard operative view with conventional light since the NIR spectrum is not visible with the human eye. ICG can be cost-prohibitive, with imaging systems costing well over $100,000 [55]. There have also been no substantial clinical trials assessing its effect on extent of resection and patient outcomes.

### 2.4. Future Targets

As fluorescein and ICG are non-specific markers of BBB breakdown and not markers of tumor tissue, ongoing investigation has focused on targeted fluorescence agents for higher precision fluorescence. 

Tozuleristide (BLZ-100), also known as “Tumor Paint” is a conjugate molecule of ICG and the tumor-specific peptide chlorotoxin. Extracted from scorpion venom, chlorotoxin binds to cell-surface targets on both low- and high-grade glial tissue [60]. Early clinical studies have demonstrated safety [61], and there are ongoing clinical trials assessing extent of resection and progression-free survival in malignant brain tumors in both adult and pediatric populations (NCT02234297 and NCT02462629, respectively).

Alkylphosphocholine analogs (APCs) are synthetic phospholipid ether molecules that selectively target tumors via overexpressed lipid rafts, and are retained for prolonged periods of time in the tumor microenvironment due to their resistance to catabolic breakdown [62]. In a preclinical study, two APCs, CLR1501 and CLR1502, showed a tumor-to-brain fluorescence ratio similar to 5-ALA [63]. Originally developed for PET imaging and targeted radiation, APCs may serve a future role as a surgical adjunct as well as radiotherapy for treatment, depending upon the conjugated radiolabel used.

The epidermal growth factor receptor (EGFR) has been commonly found to be overexpressed in glioblastoma, and as a result, is a target for antibodies and peptides [64]. Cetuximab-IRDye 800, represents a new NIR agent that can target EGFR. Conjugation of the EGFR inhibitor, cetuximab, with a NIR fluorophore, has recently been found to be safe and effective in distinguishing tumor in both contrast-enhancing and non-contrast-enhancing tumor regions with a good signal to noise background [65]. ABY-029, another EGFR inhibitor conjugated with IRDye800, has also been shown to be safe for human use in preclinical studies [66]. 

## 3. Image Guidance

Various imaging modalities, including neuronavigation, intraoperative MRI (iMRI), ultrasound and most recently the exoscope have been studied to further improve intraoperative visualization and detection of brain tumors. Each modality is described below, and corresponding landmark studies are summarized in Table 2. 

### 3.1. Neuronavigation

The use of neuronavigation has become a common and routine technology utilized in neurosurgical oncology. Neuronavigation provides the neurosurgeon guidance in localization of tumor tissues and eloquent regions of the brain. However, since preoperative imaging is used to register most neuronavigation systems at the start of surgery, brain shift during actual tumor resection can render neuronavigation systems inaccurate. There has only been one randomized controlled trial conducted assessing the use of neuronavigation on patient outcomes, which found no differences in residual contrast-enhancing tumor, and a shorter median survival in the navigation group [67]. While neuronavigation has not proven itself as a sole adjunct, it has been attributed to increasing surgeon confidence and safety, as well as allowing for smaller craniotomies. [76]. Additionally, neuronavigation has been shown to improve resection and survival when combined with iMRI in multiple prospective studies [77,78].

### 3.2. Intraoperative MRI

Since the 1990s, neurosurgeons have brought MRI technology into the operating room to improve neuronavigation and maximize the extent of resection of brain tumors [79,80]. Intraoperative MRI (iMRI) can assist surgeons in demarcating the limits of resection in relation to eloquent or critical brain structures, and in deciding if the goals of surgery have been achieved. iMRI can permit re-registration of neuronavigation systems to account for brain shift during surgery. To enhance functional navigation, iMRI can also permit diffusion tensor imaging (DTI), allowing for preservation white matter connections such as the corticospinal tract while maximizing the extent of resection [81]. In 135 glioblastoma patients who underwent resection, Kuhnt et al. found that residual contrast-enhancing tumor was identified by iMRI in 65% of cases, leading to additional resection in 19 cases [82]. In almost half of these cases, EOR was improved to a GTR with further resection of contrast enhancing tumor tissue. Patients who received a GTR (EOR ≥ 98%) had a mean OS of 14 months, compared to nine months in those who did not [82].

Improved six-month PFS and overall survival rates with iMRI have been demonstrated in other non-randomized studies. The strongest evidence to date on the efficacy of iMRI in glioma surgery outcomes is a randomized controlled trial by Senft et al. where 58 patients were randomized to conventional surgery with or without iMRI use. The EOR (96% vs. 68%, *p* = 0.023) in the iMRI group was significantly higher, and patients who had a GTR experienced better outcomes with longer PFS [68]. In a recent multicenter study, iMRI increased tumor EOR (78.4% vs. 72.7% in patients with intended GTR) and the GTR rate from 30.7% to 71.5% postoperatively. iMRI was a significant predictor of GTR on multivariate analysis. However, iMRI was not an independent predictor of overall survival [83].

Despite the significant advantages of iMRI capability, it does have limitations, mostly in its feasibility and cost. iMRI is not widely available due to extensive operating room infrastructure requirements and expertise required to carry out its use. Additionally, iMRI has been found to increase the operative time by approximately one hour [84], which may potentially pose greater intraoperative risk to patients due to prolonged anesthesia time [85]. However, iMRI has been shown to be cost-effective in the treatment of HGG, showing an incremental benefit of 0.18 quality-adjusted life years (QALYs), making the argument for a wider adaptation of the technology [86]. 

### 3.3. Intraoperative MRI and 5-ALA

Combined intraoperative MRI and 5-ALA FGS may have advantages over either adjunct alone. In two studies, one prospective cohort and one a retrospective case-control study, iMRI plus 5-ALA has been shown to yield GTR (defined as complete resection of contrast-enhancing tumor (CRET) in one study, EOR > 95% in the other) rates of 45–100% in lesions amenable to complete resection [72,73]. Additionally, in a study aimed at supratotal resection beyond the contrast-enhancing tumor, Eyupoglu et al., found that patients who underwent iMRI plus 5-ALA had a longer overall survival compared to patients who underwent iMRI alone (18.5 vs. 14 months, *p* < 0.0001) [87]. In a comparative study, 5-ALA was found to be both higher in sensitivity and specificity in detecting pathological tissue at the infiltrative margin [88], and Roder et al., found a higher rate of GTR in patients undergoing iMRI versus those who received 5-ALA [69].

### 3.4. Exoscope

While the conventional microscope has stood the test of time as the mainstay for visualization during glioma surgery, the exoscope has been introduced into neurosurgery as a visualization device for the resection of brain tumors. The exoscope permits the neurosurgeon and the operative team to visualize the surgical area on a high-definition heads-up display. There are several advantages of the exoscope that have made it a favorable alternative to the conventional microscope. The exoscope provides approximately double the optical zoom of the microscope and employs light-emitting diode (LED) lighting, which reduces tissue glare, the risk of thermal damage to tissue, and may delineate tumor tissue better [89]. Recently, exoscope visualization technology and patient outcomes have been studied in glioblastoma tumors, in a cohort of 26 patients with mostly eloquent tumors. The use of a robotic-assisted exoscope in combination with neuronavigation that incorporated diffusion tensor tractography (DTI) for eloquent pathway visualization, permitted for large EOR (over 78%) of contrast-enhancing tumor tissue and six-month PFS in 86% of patients [90]. These findings are comparable to recent studies on GBM surgery using other adjuncts [69,71,83]. Combined use of the exoscope and fluorescence-guided surgery is currently under active investigation.

### 3.5. Intraoperative Ultrasound

Intraoperative ultrasound (IOUS) is inexpensive, widely available, and provides real-time visualization of tumor. It can be integrated into neuronavigation systems to compensate for brain-shifts that may render neuronavigation inaccurate [91]. IOUS has been shown to have high sensitivity and specificity both adult and pediatric brain tumors [92]. Ultrasound is a highly multi-modal technology, and may be used to determine tissue composition with elastosonography [81], detect flow patterns in tumor vessels with micro vessel power doppler [93] and may be fused with MR imaging to reduce radiation with fluoroscopy [94].

Contrast-enhanced ultrasound (CEUS) is a contrast-specific imaging modality that allows for better delineation of tumors compared to conventional ultrasound [95]. Over the past decade, CEUS has been studied, with the advantage of providing Doppler to visualize areas of increased vasculature and perfusion [95]. Prada et al. found that in all 10 cases of glioblastoma resection, additional tumor was found using CEUS and was confirmed with histopathology [96]. In a retrospective review of 76 patients with glioblastoma, Neidert et al. found an increase in overall survival (21.9 vs. 18.8 months) and PFS (7.1 vs. 3.4 months) with intraoperative ultrasound (iUS) compared to the non-iUS group [74]. 

### 3.6. Intraoperative Mapping and Neuromonitoring

#### 3.6.1. Intraoperative Mapping

Intraoperative mapping has been established as the gold-standard for identifying eloquent brain tissue during tumor surgery [97]. Stimulation mapping with bipolar or monopolar stimulation in the cortical and subcortical tissues can sensitively detect motor, language, and other eloquent pathways during surgery [98]. While operating in and around functional territories, stimulation mapping, assists surgeons in identifying safe corridors of access to tumors and provides real-time feedback on the proximity of the resection cavity to critical structures [99]. However, mapping can be limited by the presence of preoperative neurological deficits, such as hemiparesis and dysphasia, and the ability of the patient to cooperate during awake surgery. Combined with 5-ALA, intraoperative mapping has been shown to be a useful adjunct in eloquent region surgery, by enabling complete resection in up to 96% of patients, with minimal postoperative neurological declines [100,101]. Awake mapping combined with iMRI may also provide benefit in eloquent region resections [102,103]. 

#### 3.6.2. Intraoperative Neurophysiologic Monitoring (IONM)

While prior studies have not identified a patient survival advantage with the use of neuromonitoring, monitoring for functional preservation has remained a primary objective with the use of IONM [104]. Despite previous criticisms of false-negative motor evoked potential (MEP) monitoring affecting IONM reliability, previous studies have shown reliability in MEP monitoring, with few to no false-negative results [105,106]. 

### 3.7. Intraoperative Histopathology and Imaging Probe Devices

Since the era of Harvey Cushing, understanding the histopathology of a central nervous system tumor in the operating room has been crucial in guiding surgery [107]. Intraoperative histopathological data provides the surgeon information regarding the level and type of malignancy, which is an important factor in surgical decision-making. Conventionally, an intraoperative pathological diagnosis requires frozen sectioning, cytological preparations, and technicians and pathologists available in real-time to interpret slides. This labor-intensive system creates delays in clinical decision-making and increases operative time. 

### 3.8. Raman Microscopy

In 2008, the creation of stimulated Raman scattering (SRS) microscopy allowed for high-resolution imaging of label-free, unprocessed tissue [108]. Since then, SRS has been used over the past decade to diagnose various types of cancers [109]. Orringer et al. were able to engineer an SRS microscope that was employed in the operating room, to process imaging of tissues acquired at surgery and simulate hematoxylin and eosin (H & E) staining, called stimulated Raman histology (SRH) [110]. Additionally, Orringer et al. were able to create an algorithm that could use this technology to predict tumor subtypes with 90% accuracy. Handheld probes using Raman spectroscopy have pushed the envelope of what can be visualized in the operating room, by being able to detect infiltrative cells at tumor margins [111], and differentiating high- and low-density tumor regions [112]. Artificial intelligence (AI) has further permitted the actual diagnosis of tumor tissues with use of the SRS system, with diagnostic abilities non-inferior to pathologist-based interpretation [113].

#### 3.8.1. Probe-Based Microscopy

Handheld probe devices have been developed to further aid the neurosurgeon in detecting tumor tissue in the resection cavity. While certain patient factors such as tumor molecular signatures and MGMT methylation may portend prolonged survival [114], as prior studies have shown, extent of resection does matter, with differences in time to tumor progression and overall survival seen between gross and near-total resection [115]. For this reason, probes are being developed to detect tumor tissue at the cellular level. These probes can combine lasers, lenses and filters, connected with a camera and spectrometer to provide better detection and visualization of tumor cells, while not adding hindrance to operating room conditions for surgeons [116,117,118].

Several studies have applied handheld Raman spectroscopy to the operating room setting. In a case series of patients undergoing resection of WHO grade II–IV gliomas, Jermyn et al. concluded that Raman imaging allowed more accurate detection of cancer cells compared to white light microscopy and MRI [119]. Using a detection system that combines intrinsic fluorescence spectroscopy, diffuse reflectance spectroscopy, and Raman spectroscopy, Jermyn et al. and others have detected brain, lung, colon, and skin cancers with 97% accuracy and 100% sensitivity [120,121,122]. While other technologies have focused on improving visualization of the contrast-enhancement regions of tumors on MRI, Raman spectroscopy allows for distant visualization of tumor cells, beyond what T1 post-contrast and T2 imaging can detect, which may translate to prolonged survival [123]. Handheld confocal microscopy has also been shown to detect and quantify PPIX fluorescence at the cell level in gliomas [124,125]. 

While visually inspecting the tumor architecture with microscopy is useful, providing a quantitative assessment of the tumor-cell density of the region of interest has the potential of permitting the neurosurgeon to maximize extent of tumor resection at the cellular level. Handheld probes have been shown to provide greater sensitivity of 5-ALA detection in both low and high-grade gliomas [126,127,128]. This increased sensitivity is limited, however, by the small surface area of detection, which is difficult to assess in a large resection cavity. Quantitative spectroscopy is also limited by its processing speeds, and ongoing research is focusing on improving speed and precision of fluorescence detection, to allow for surveillance of larger resection cavities [129].

#### 3.8.2. Wide-Field Endomicroscopy

To address the challenge of tumor margin assessment in constrained operative corridors, the use of a modified endoscope has been explored. By placing the endoscope tip near tumor tissues in difficult to visualize corridors, blind spots may be more easily visualized. Multiple studies have combined PPIX fluorescence from 5-ALA and fluorescein with endomicroscopy to detect and quantify fluorescence [130,131,132,133]. In a study of 74 patients who underwent resection with laser endomicroscopy and fluorescein sodium, there was a sensitivity and specificity for gliomas of 91% and 94%, respectively [17]. Additionally, confocal laser endomicroscopy has been shown to be not only effective in high-quality visualization, but ergonomically friendly to surgeons with ease of use [134]. 

## 4. Summary

**Figure 4:**
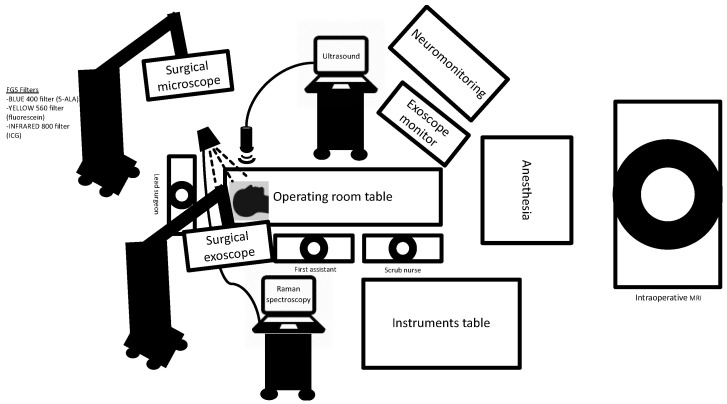
Operating room schematic outlining the various surgical adjuncts used in malignant glioma surgery, including fluorescence-guided surgery, visualization with the surgical microscope and exoscope, Raman spectroscopy with handheld probe devices, intraoperative ultrasound, intraoperative MRI and neuromonitoring.

Technological advancements have provided neurosurgeons with a plethora of surgical adjuncts to maximize the resection of high-grade gliomas, as seen in the schematic outlined in Figure 4. Maximal cytoreduction has been associated with better patient outcomes in glioma surgery. Supported by a strong body of evidence, both fluorescence-guided surgery and intraoperative image guidance have been adapted by many neurosurgeons and have now become standard of care in the operating room. Newer visualization technologies such as the exoscope may permit greater magnification and delineation of tumor tissue. Microscopy techniques, such as SRH, have made significant strides over the past decade, and for the first time, surgeons are able to evaluate the tumor architecture at a microscopic level in the operating room. Handheld devices may also permit resection down to the tumor cellular level to further push the limits of EOR. All of the surgical adjuncts discussed are not mutually exclusive, and as previous studies have shown, may be more effective in improving surgical outcomes when combined. Surgeons must select adjuncts to employ based on tumor characteristics and their own experience with the various technologies. It is possible that an adjunct with clear benefit for one case may not be useful or appropriate for another. Furthermore, the patient’s safety and preservation of neurologic function must be the goal of every surgery. While some technologies are more resource-intensive than others, what has been established is that the bar has been raised in neurosurgical oncology with the surgical adjuncts available. Safety and maximal extent of tumor resection are no longer merely the goal, but the expectation. 

## Figures and Tables

**Table 1:** Fluorescence-guided surgery for high-grade glioma evidentiary table.

Author (Year)	Fluorophore	Study Design	Evidence Class	Description	Conclusions
Stummer et al. (2000) [6]	5-ALA (PpIX)	Case series	II	Prospective study of 52 patients receiving 5-ALA for GBM resection. Rates of complete resection and predictors of survival were assessed.	Complete resection of contrast-enhanced tumor was achieved in 63% patients. Age, residual fluorescence and absence of contrast-enhancement on postoperative MRI were predictors of survival.
Stummer et al. (2006) [7]	5-ALA (PpIX)	RCT	I	Randomized, controlled multicenter phase III trial of 322 patients who received either 5-ALA or conventional surgery. EOR and PFS were analyzed.	There was a significant improvement in complete resection of contrast-enhancing tumor in the 5-ALA group (36% vs. 27%), and improved six-month PFS (41.0% vs. 21.1%).
Eljamel et al. (2008) [8]	5-ALA (PpIX)	RCT	I	Randomized, prospective phase III single center trial evaluating the use of 5-ALA and repetitive photodynamic therapy (PDT) for the treatment of GBM. Survival, Karnofsky performance score (KPS) and time to tumor progression were analyzed.	Patients who received 5-ALA and PDT had a significantly prolonged survival (53 vs. 25 weeks), improved KPS and prolonged time to tumor progression (8.6 vs. 4.8 months) compared to controls.
Nabavi et al. (2009) [9]	5-ALA (PpIX)	Case series	II	Multicenter, prospective study of 36 patients with HGG undergoing surgery with 5-ALA. Positive predictive value (PPV) and survival data was analyzed.	5-ALA had over 90% PPV in both areas of strong (96.9%) and weak (90.3%) fluorescence. No adverse events were found using the drug.
Diez Valle et al. (2011) [10]	5-ALA (PpIX)	Case series	II	Prospective, single-center study of 36 patients with GBM who received 5-ALA prior to surgery. EOR, complete resection of contrast-enhanced tumor and survival analysis was conducted.	Strong fluorescence yielded 100% PPV, while vague fluorescence beyond the tumor core yielded 97% PPV and 66% negative predictive value (NPV). Complete resection of the contrast-enhanced tumor was removed in 83.3% patients. Patients had a 8.2% morbidity rate one month after surgery.
Stummer et al. (2011) [11]	5-ALA (PpIX)	Case series	II	Prospective, multicenter phase II safety trial assessing adverse events in 219 patients undergoing HGG resection with 5-ALA who were also receiving concomitant radiochemotherapy with adjuvant temozolomide (Stupp protocol). Adverse events (AE) and survival analysis were conducted.	Three patients experienced four AEs possibly related to 5-ALA. GBM patients experienced a survival advantage if they received radiochemotherapy (16.3 vs. 11.9 months). Elderly patients additionally saw a benefit from concomitant therapies.
Diez Valle et al. (2014) [12]	5-ALA (PpIX)	Cohort	II	Retrospective, multicenter study of 251 patients with malignant glioma who received 5-ALA with intended chemoradiotherapy with temozolomide. Complete resection rates and survival analyses were conducted.	Rates of complete resection (67% vs. 45%) and six-month progression-free survival for GBM patients (69% vs. 48%) were significantly higher in the 5-ALA group.
Eljamel (2015) [13]	5-ALA (PpIX)	Meta-analysis	II	Meta-analysis of 20 studies on the use of 5-ALA for GBM surgery. Outcomes parameters included GTR rates, time to tumor progression, overall survival, and sensitivity and specificity data.	Mean GTR rate was 75.4%, and mean time to tumor progression was 8.1 months. Mean overall survival gain was 6.2 months. Mean specificity was 88.9% and sensitivity of 82.6%. 5-ALA is highly sensitive and specific, and improves GTR and time to tumor progression.
Teixidor et al. (2016) [14]	5-ALA (PpIX)	Cohort	II	Prospective, multicenter cohort study of 85 patients with HGG receiving 5-ALA prior to surgery. Safety data, EOR and survival analyses were conducted.	Complete resection was achieved in 54% of patients. Six-month PFS was 58% and median overall survival was 14.2 months. No serious adverse events were reported. One-month postoperative morbidity was 6.5%.
Koc et al. (2008) [15]	Fluorescein	Cohort	II	Prospective study of 80 patients with GBM, 47 who received fluorescein during surgery and 33 who did not. EOR and survival analyses were conducted.	Patients who received fluorescein were more likely to receive a GTR, however, there were no differences in median survival between groups.
Acerbi et al. (2014) [16]	Fluorescein	Case series	II	Prospective study of 20 patients with HGG who received fluorescein during surgery. Safety data, EOR and survival analyses were conducted.	No adverse events related to fluorescein were observed. Complete removal of the contrast-enhanced tumor was found in 80% patients. Six-month PFS was found in 71.4% of patients, and median overall survival was 11 months.
Martirosyan et al. (2016) [17]	Fluorescein	Case series	II	Prospective, single-center study of 74 patients with gliomas and meningiomas who received fluorescein during surgery and resection with confocal laser endomicroscopy. Sensitivity and specificity data were analyzed.	Sensitivity and specificity for glioma tissue was 91% and 94%, respectively.
Acerbi et al. (2018) [18]	Fluorescein	Case series	II	Prospective, multicenter phase II trial of 46 patients with HGG who underwent resection. EOR, PFS and overall survival was recorded.	82.6% gross total resection, PFS-6 and PFS-12 were 56.6% and 15.2%. Median survival was 12 months. No adverse reaction related to SF administration was recorded. The sensitivity and specificity of fluorescein in identifying tumor tissue were respectively 80.8% and 79.1%.
Cho et al. (2020) [19]	ICG	Case series	II	Retrospective study of 36 patients with HGG who received ICG prior to surgery. Accuracy of fluorescence was analyzed.	Near-infrared (NIR) imaging showed higher sensitivity and accuracy in diagnosing HGG tissue intraoperatively compared to white light. NIR imaging predicted postoperativce MRI gadolinium contrast with 91% accuracy, and patients with no residual NIR signal following resection were more likely to have complete resection on postoperative MRI.

5-ALA, 5-aminolevulinic acid; PpIX, protoporphyrin IX; RCT, randomized controlled trial; GBM, glioblastoma; MRI, magnetic resonance imaging; EOR, extent of resection; HGG, high-grade glioma, GTR, gross total resection; ICG, indocyanine green.

**Table 2:** Intraoperative image guidance for high-grade glioma evidentiary table.

Author (Year)	Modality	Study Design	Evidence Class	Description	Conclusions
Willems et al. (2006) [67]	Neuronavigation	RCT	I	45 patients randomized to surgery with or without neuronavigation. Residual contrast-enhancing tumor and survival data was analyzed.	There were no differences in residual contrast-enhancing. Median survival was shorter in patients who received neuronavigation.
Senft et al. (2011) [68]	iMRI	RCT	I	58 patients randomly selected to iMRI or control for glioma surgery. Extent of resection and postoperative neurological deficits were analyzed.	Patients in the iMRI group had higher rates of complete tumor resection, and no increased postoperative neurological deficits.
Roder et al. (2014) [69]	iMRI + 5-ALA	Case series	II	Retrospective comparative study of 117 patients undergoing GBM surgery with iMRI compared to conventional surgery with and without 5-ALA.	iMRI patients had a lower residual tumor volume and higher proportion of complete resection. Improved six-month PFS was seen in cases of complete resection.
Kubben et al. (2014) [70]	iMRI	RCT	I	Randomization of 14 patients with supratentorial GBM received iMRI or conventional neuronavigation. Residual tumor volume and postoperative outcomes were calculated.	There were no differences found in residual tumor volume or median survival. iMRI did not appear to be cost-effective, but limited by a small patient sample.
Wu et al. (2014) [71]	iMRI	RCT	I	114 patients were randomized to iMRI or conventional neuronavigation. EOR was the primary endpoint, with secondary endpoints survival and morbidity data.	No differences in rates of GTR were detected in HGG patients. Six-month PFS trended toward the iMRI group in HGG patients.
Coburger et al. (2015) [72]	iMRI + 5-ALA	Case series	II	Prospective trial of 33 patients undergoing GBM surgery with iMRI and 5-ALA, compared to retrospective controls, EOR and survival data was analyzed.	EOR was higher in the iMRI+5-ALA group compared to iMRI alone. There were no differences in postoperative neurological deficits or survival data between groups.
Schatlo et al. (2015) [73]	iMRI + 5-ALA	Case series	II	Retrospective series of 200 HGG patients undergoing surgery with iMRI and 5-ALA or conventional surgery. EOR and survival data was analyzed.	Patients in the iMRI + 5-ALA group experienced prolonged overall survival upon univariate analysis, but no differences were detected upon multivariate analyses.
Neidert et al. (2016) [74]	Ultrasound	Case series	II	Retrospective analysis of 76 patients who underwent glioblastoma resection with intraoperative ultrasound or conventional surgery. Only patients who had a GTR achieved were included. Survival data was analyzed.	Median overall survival was longer in GTR patients were ultrasound was used, and ultrasound was associated with prolonged overall and progression-free survival.
Golub et al. (2020) [75]	iMRI + 5-ALA, Neuronavigation	Meta-analysis	II	Meta-analysis of 11 studies assessing neuronavigation, iMRI and 5-ALA for HGG resection. Rates of GTR and survival comparisons were analyzed.	iMRI and 5-ALA were superior to neuronavigation in achieving GTR, and both modalities were shown to improve patient survival. However, no differences were found between iMRI and 5-ALA.

## Data Availability

The data presented in this study are openly available in Pubmed at https://pubmed.ncbi.nlm.nih.gov/.
